# Insights gained through exploring UK midwifery care by US midwifery graduate students in a short-term study abroad: A qualitative study

**DOI:** 10.18332/ejm/192929

**Published:** 2024-10-07

**Authors:** Cindy L. Farley, Jalana Lazar, Debora Dole

**Affiliations:** 1 Nurse-Midwifery/Women’s Health Nurse Practitioner Program, School of Nursing, Georgetown University, Washington, United States; 2 School of Nursing, Georgetown University, Washington, United States

**Keywords:** short-term study abroad, midwifery-led units, midwifery education, cultural humility, professional role development, midwifery student confidence

## Abstract

**INTRODUCTION:**

Student midwives exposed to effective systems of midwifery care in other countries can consider how aspects of this knowledge can translate into their healthcare system to improve maternal and infant perinatal outcomes. An optional short-term study abroad (STSA) experience was developed for US midwifery graduate students to expose them to the UK healthcare system, where midwives are considered the primary professionals for the care of the childbearing family. This qualitative study explored the influence of an STSA experience on US midwifery graduate students’ learning of midwifery in the UK.

**METHODS:**

Ten midwife student participants wrote pre- and post-trip narratives in the US and daily diary entries during their week in the UK. A grounded theory approach guided the content analysis. The themes were derived from the NVivo software data by three midwife researchers who value global health learning experiences. Analysis was shared with participants to ensure its trustworthiness.

**RESULTS:**

Themes that emerged included: ‘Another viewpoint’, encapsulating curiosity and comparison of US and UK midwifery; ‘Eye-opening’, capturing surprise at noted differences between US and UK midwifery practice; and ‘Goals met and influenced’, expressing how their learning is anticipated to shape their professional identities and career trajectories going forward.

**CONCLUSIONS:**

US student midwives exposed to functional systems in countries where midwifery care is fully integrated, broadened their views of midwifery care and practice. They became inspired to make positive changes in the US. Educational opportunities for midwifery students, such as STSA experiences, can positively influence self-confidence and professional identity.

## INTRODUCTION

Midwifery care is associated with improved maternal and infant outcomes^1^. Maternal morbidity and mortality rates in the US have steadily increased, with Black and Brown childbearing people experiencing higher rates of morbidity and mortality, even in well-resourced areas of the country^2^. Restrictive policies, hostile work environments, and lack of midwifery leadership and voice in administrative decisions limit the ability of midwives to apply their expertise in supporting physiologic birth. Midwifery educators are challenged to create educational opportunities that instill knowledge and inspire activism for positive change. Exploring other countries with better perinatal outcomes, such as the UK, that use midwives as the primary care providers for childbearing people, can encourage such change. This article describes a short-term UK study abroad (STSA) experience developed by faculty teaching in a US university designed to prepare midwife champions for change.

The US ranks worst among 16 comparable high-income countries in their perinatal morbidity and mortality indices but ranks highest in per capita spending on healthcare^3,4^. Of particular interest to this STSA developed to the UK, selected US to UK comparisons during 2019 (the time of travel) were made. The US compares to the UK in the following indices: maternal mortality (20.1 to 6.5), infant mortality (5.6 to 3.7), and per capita spending on health care ($11582 to $3055)^2-8^. The reasons for the standing of the US are multi-faceted and complex, yet some strategies that can improve these outcomes are underused in the US. The widespread use of midwives supporting physiological birth is one such underused strategy^9^.

Midwifery in the US is growing but still small in numbers and impact. The numbers of midwives in the general population in the US compared to the UK during the 2019 STSA experience were 13000 to 329 million people and 47000 to 67 million, respectively^6,10^. US midwives need to carve out space within existing healthcare structures to support physiological birth. Midwife-led units (MLUs) located in hospitals are proposed as a solution for improving maternity care in the US. MLUs are common in the UK and uncommon in the US^11^. MLUs offer an opportunity for midwives to practice to the full extent of their education and expertise with timely access to physician-collaborative care for complex conditions. Champions are needed to carry this work forward in existing US systems^12^. Graduate midwifery students exposed to functional MLUs in other countries can be inspired to champion such change in the US.

STSAs allow learners to immerse themselves in a different culture, providing real-world contexts for learning and fostering personal growth^13^. Experiential learning theory emphasizes the importance of concrete experiences and reflective observation. STSAs encourage participants to engage directly with the host culture and observe and experience differences. Educators creating STSAs support transformative learning experiences that empower adults to expand their cultural sensitivity, humility, critical thinking skills, and global perspectives^14^.

Little has been published about the impact of STSA experiences on midwifery students. One qualitative study examined the intentions of Irish midwifery students to study abroad and found that enhanced professional identity, cultural sensitivity, and employability emerged as moderating factors^14^. Language and cost were also mentioned. Another qualitative study explored the longer term impacts of STSAs and found that such participation can be transformative and positively influence personal and professional development^15^. Two literature reviews of international experiences on pre-registration nursing and midwifery students found an impact on cultural learning, personal growth, and professional development. However, the authors of both reviews noted a lack of research specific to midwifery students^16,17^. This qualitative study answers the research question: ‘What substantive and affective learning occurred in US midwifery students about UK organizational structures and midwifery practice in an STSA experience?’. The learning of US students participating in an STSA experience in the UK in the final term of their midwifery studies is described.

## METHODS

### Study design and setting

Qualitative methods were well-suited to address the research question. A grounded theory approach to content analysis was used to examine the cognitive and affective learning of the students participating in the STSA, whose itineraries included 2.5 days in London and 2 days in Oxford, England (Table 1).

**Table 1:** Itinerary of a short-term study abroad (STSA) to the UK in 2019, of midwifery students of Georgetown University, USA (N=10)

*Day*	*Events*
**Pre-trip travel**	Students made individual arrangements Some added a few days of personal travel ahead of the STSA Students arranged an evening cruise on the Thames
**Day 1**	Lecture, tea and biscuits with Royal College of Midwives leader PhD in Midwifery candidate presented research on induction options Tour of London area hospital’s MLU, OBU and clinics British pub dinner and sharing with local midwives
**Day 2**	Sharing and Q&A with midwifery faculty and students at a London university Two PhD in Midwifery candidates presented their research Tour of an East London FMU Return to London via water taxi on the Thames Free time from mid-afternoon on
**Day 3**	Joined 1st year UK midwifery students in their ethics lecture Took a train from St. Pancras to Chatham Historic Dockyards for tour of Call the Midwife filming locations, high tea included Travel to Oxford
**Day 4**	Small group sharing among 2nd and 3rd year UK students and US students Lectures by UK and US faculty Tours of Oxford area AMU and OBU
**Day 5**	Values reflection with Jesuit leader at University of Oxford Tour of Cotswold village FMU Group debrief of the experience
**Post-trip travel**	Students made individual arrangements

MLU: midwife-led unit. OBU: obstetrics unit. FMU: freestanding midwifery unit.

### Participants

US students eligible for travel were those in the final term of their midwifery education program at Georgetown University in the Fall 2019. Students were sent e-mail invitations to participate. Additionally, the researchers were available to answer questions through dialogue and e-mail. The STSA experience included interaction with UK midwives, professors, and students who were not participants in the study.

Preparation for the STSA included required readings on MLUs, global engagement, and travel safety^18,19^. Each traveling student chose a unique care practice to compare US and UK clinical practices, such as vaginal breech birth and breastfeeding initiation. Hosting and visiting students volunteered to connect as international internet pen-pals prior to travel to establish cross-country conversations and develop personal connections.

### Data collection

Participants wrote pre- and post-trip narratives in the US and completed daily diary entries in the UK. Their writings were the data for this study. Pre-trip prompts asked for responses to the following questions: ‘How do MLUs compare to your knowledge and experience of US models of midwifery care?’, ‘What questions do you have for UK midwives and students?’, and ‘How might this STSA experience help you, as you embark on your career in the US?’. Daily diary prompts were: ‘For each day of your trip, describe objectively what you did and saw that day’; ‘Reflecting on your activities, how did you feel?’, and ‘What learning did you gain from it?’. Post-trip prompts included: ‘Did you meet your learning goals for this experience?’, ‘Describe how this experience did or did not support your learning goals’; ‘How might this STSA experience inform the development of your role as a midwife?’, ‘How has this experience influenced your understanding of the organizational structure and leadership qualities necessary to sustain midwifery-led practice in the US?’, and ‘How has this experience influenced your future career goals?’.

Pre- and post-trip narratives were 2–3 pages in length. Daily diary entries were 1–2 pages daily, and some included photographs. Files were received from the students and placed in a secure university cloud storage platform with encryption and password protection.

### Ethical considerations

Ethical approval for this study (00001159) was granted by the Georgetown University Institutional Review Board on 27 August 2019. Participation in the study was not required to participate in the STSA. Criteria for the study were verbally explained, and consent to participate was obtained in writing. These students were not in the researchers’ classes during the STSA and data collection; thus, potential risks of student vulnerability were mitigated. Students understood this experience was optional and required arranging travel, school, and personal obligations to participate.

### Data analysis

The expressed meaning of this experience was elucidated via the texts produced by the participants. The three researchers created an initial coding tree using NVivo 12 software derived from the data. Secondary categories were developed as patterns emerged and reconstructed into themes to provide deeper understanding of this experience from the students’ perspective. Quotes were de-identified and indicated by letter. The data analysis and emerging themes were shared with participants to ensure the trustworthiness of the analysis. All students reported support for the findings.

## RESULTS

Thirteen of 23 (56%) eligible students expressed interest in the trip, and ten (43%) were able to travel. Those unable to participate faced personal situations that prevented travel. Of these three, two were male midwife students. Travelers identified as women were registered nurses with a Bachelor’s degree in the final term of their midwifery education program. Seven students were White, 3 were Black, and one was Asian. They ranged in age from mid-20s to mid-30s.

**Figure 1:**
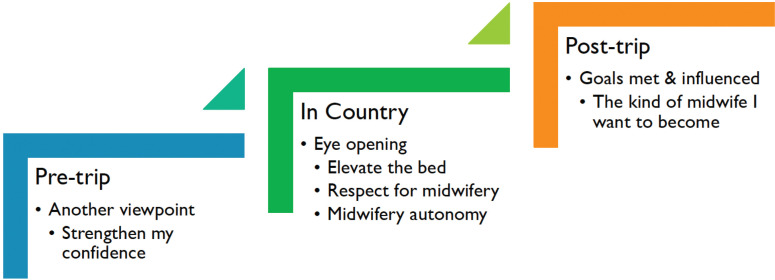
Content themes by time period

A logical temporal organization of themes emerged from the content analysis, reflecting evolving learning by the US students (Figure 1). The pre-trip theme ‘Another viewpoint’ demonstrated anticipation and curiosity with a subtheme of ‘Strengthen my confidence’, indicating the importance of affective learning. The STSA in-country theme was ‘Eye-opening’, which comprised the subthemes: ‘Elevate the bed’, ‘Midwifery respect’, and ‘Midwifery autonomy’. The concluding theme was ‘Goals met and influenced’, encompassing post-trip reflections and a subtheme on early conceptualizations of midwifery identity: ‘The midwife I want to become’.

### Pre-trip themes

*Theme: Another viewpoint*

British midwifery is a positive example of midwifery care integrated into a national healthcare system. US students were eager to gain valuable insights from their UK counterparts. In preparation for this trip, one student reflected on her formal learning journey in becoming a midwife:

*‘At first, [midwifery] was knowing I could have the option to complete the labor experience I just had with this woman and catch her baby, and that would be all. But now I see so much more of the potential impact and difference I could and want to make. I truly want to learn from a country that has much better outcomes than [the US]. I want to know what I can do to make a difference.’* (#F)

Another student encapsulated the intent of this STSA in her pre-trip preparation:

*‘This experience in England will provide … another viewpoint of midwifery care in another first-world country. Midwives in America are not [in] abundance, and the maternal mortality rate is rising with an increased distrust in obstetric providers.’* (#D)

Students’ own learning and clinical experiences to date gave them points of comparison for their UK STSA.

*Subtheme: Strengthen my confidence*

Students mentioned building confidence as an expected feature of the STSA:

*‘My time abroad will hopefully strengthen my confidence as a midwife. I hope to learn more about the basic integration of midwives into the everyday care of women.’* (#A)

This subtheme underlines student recognition of the emotional aspects of becoming a midwife:

*‘Learning about other evidenced-based practices … that are being utilized daily will help to bolster my confidence in being able to provide women with quality care. Having heard so many great things about midwifery in the UK and reading how well-respected the profession is there has really made me feel empowered.’* (#B)

Confidence in their future midwifery practice was undermined for some students by the US maternity care system, which can be challenging. One student reflected:

*‘The lack of support also makes it harder for midwifery to build confidence in practicing full midwifery care in the hospital setting for low-risk pregnancies.’ (#J)*

One student’s unique familial connection to UK midwifery education and practice fueled her desire for this STSA experience:

*‘As a third-generation future midwife, it has always been a dream of mine to go to London and learn midwifery where my mother was taught.’* (#G)

This student struggled with the dichotomy of the medicalized births she saw in US hospitals compared to the stories of physiological home births she heard from her grandmother and mother:

*‘My mother always told me “This is not how we practiced in London”. She always made it very clear that birth in the UK was powered by midwives confident in their ability to bring babies earthside without interventions. I believe this trip will help me restore my confidence in birth and learn new techniques for assisting women in labor.’* (#G)

### In-country themes

Once in the country, students were fully engaged in the days’ events. Experiential learning involves one’s senses and emotions; this came through in the language used by US students in their daily reflections. The primary theme that emerged was ‘Eye-opening’, with subthemes of: ‘Elevate the bed’, ‘Respect for midwifery’, and ‘Midwifery autonomy’.

*Theme: Eye-opening*

Differences between UK and US midwifery structure and practice were described as ‘eye-opening’. This sense of unexpected enlightenment came with varied reactions. US students compared what they saw in the UK with their US experiences. Student comparisons of US and UK midwifery care reflected specific scope and practices deemed superior for both.

During a joint class of UK and US students, selected areas of midwifery practice were discussed. These peer-to-peer comparisons of student experiences were powerful and universally endorsed as one of the most informative experiences of the trip by the US students:

*‘Students were asked to raise their hands if they had been involved in a water birth. The majority of UK students’ hands were raised.’* (#F)

The student said:

*‘This was eye-opening for me. There were even some students that have been involved in a breech delivery.’* (#F)

Few US students have the opportunity to participate in water birth, and even fewer in vaginal breech birth. One student reflected on her surprise at the availability of these practices for UK students by comparing the US midwifery scope of practice, which includes primary and reproductive healthcare for those who are not pregnant, with the UK midwifery scope of practice focused on childbearing health care:

*‘It was surprising to know how limited their [UK] scope of practice was, yet they have so much experience with advanced birth.’* (#G)

The hospital environment was noted to be different, particularly in the midwifery-led units:

*‘The MLU was life-changing, perspective changing, mindset changing. I have never seen something like that room before and am so honored to know that midwifery care like that exists within a hospital setting. It still felt like a hospital but was so much more functional than I ever thought a hospital could be for birth.’* (#E)

The US students felt the presence and leadership of UK midwives in the hospital setting:

*‘It was inspiring and eye-opening to see the different specialties that the midwives work in and to walk onto a maternity unit and see mainly midwives and only a few doctors.’* (#B)

Not all eye-opening comments were positive. For example, during a hospital postpartum ward walk-through tour, students commented on the inadvertent glimpses of patient care:

*‘The wards are like stepping back in time in the States.’* (#H)

US students are used to private hospital rooms for most childbirth care, while multi-patient wards can be seen in some UK hospitals. A student expressed dismay at this:

*‘The set-up of the UK birthing hospitals that we saw left me feeling sad for British mothers and families – while birth suites are private, the induction and postpartum areas are large, open spaces with only thin curtains for privacy.’* (#E)

Another frustration expressed by US students was the lack of understanding and curiosity about US midwifery by UK midwives and students. One student:

*‘Felt as if they have no idea what we do as midwives or labor nurses in the US and didn’t feel as if they tried to understand.’* (#E)

In the UK, maternity care workers are used instead of labor and delivery nurses. In the US, birth areas are called labor and delivery units and nurses who work there are referred to as labor and delivery nurses. Not an official title but common parlance:

*‘The UK sees midwifery and nursing very separate, but to me the work a midwife does in the UK is mostly nursing care. I did everything that UK midwives do as a labor nurse, the only thing I didn’t do was catch the baby.’* (#E)

*Subtheme: Elevate the bed*

**Figure 2:**
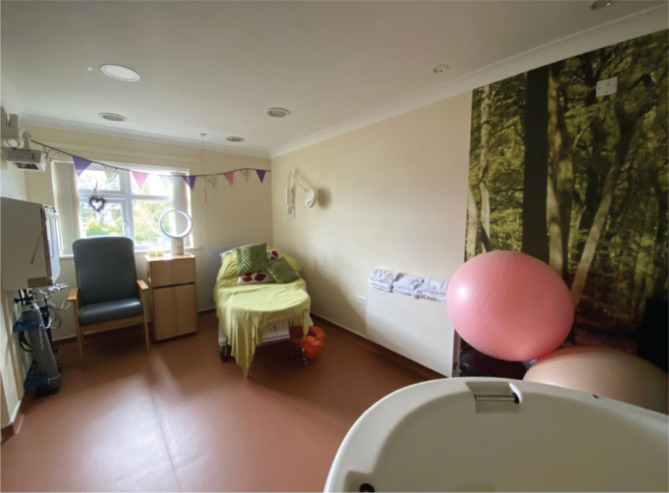
Birth room arrangement in free-standing midwifery unit; bed is not centered; birth tub is in the forefront (photo by #J)

One simple environmental change in hospitals and free-standing birth centers caught the eye of all US students (Figure 2):

*‘The midwife taught us a nice trick, which was to keep the bed elevated. Women were more likely to walk around and use other birthing apparatus if the bed is elevated. Lying in bed for long periods of time can delay labor.’* (#G)

*‘Most rooms had a huge water birthing tub, birthing chair/stool, large size bean bag chair, an elevated hospital bed (that was mostly only used for suturing).’* (#J)

Reflections included how such a change could be implemented in the US, where beds are centered in the room and where a majority of women and birthing people are confined during their labor and birth experience:

*‘The focus of the rooms on the midwife-led unit was the tub. The bed was on the side of the room and was raised all the way up to make it less attractive. I really like this technique.’* (#I)

*‘The fact that women are encouraged to not get in the bed during labor and birth is so incredible to me, and I want to implement that into my own practice.’* (#B)

Students realized that this small environmental change profoundly influenced expectations and practice:

*‘Women do not labor in bed.’* (#H)

The expectation was for laboring individuals to be upright and active or to relax in the birth tub. The option of giving birth out of bed was supported:

*‘The woman doesn’t immediately enter the room and go in the bed. The center of the attention was most definitely the large tub surround by peace-evoking backgrounds on the wall. I can see how with dimmed lights this could provide a serene environment.’* (#F)

*Subtheme: Respect for midwifery*

The respect shown for midwives and midwifery students in the UK stood out to students:

*‘I could also see how respected midwifery is in the UK almost immediately through the bulletins boards in the facilities that highlighted midwives and even students.’* (#B)

*‘I was very impressed with how much respect the student midwives were given; it felt as if they were already part of the team … or trust.’* (#G)

One student described the experience of realizing how much less respect midwifery received in the US as ‘heartbreaking’ and wrote:

*‘There is just so much respect for the midwives in the UK, and it is disheartening to know it should be this way, but it is not in the US.’* (#F)

Students spoke about how midwives and student midwives appeared to be a fundamental part of British society:

*‘In England there seems to be a spiritual understanding and respect for midwives. They are regarded as an integral part of the community.’* (#A)

Even students who preferred how midwives worked in the US expressed a desire to see midwifery as the standard of care for childbearing families like the UK. One student expressed:

*‘How grateful I am to be a midwife in the US as opposed to the UK and how much I wish midwifery was the norm in the US like it is in the UK.’* (#E)

*Subtheme: Midwifery autonomy*

The experience of witnessing UK midwifery facilities firsthand and discussing care practices with UK midwives and student midwives caused the students to reflect on what comprises an autonomous midwifery profession:

*‘It was amazing to see in action that midwives are experts in normal pregnancy and birth, and in the UK, it was normal that they were in charge of the units where the births occurred.’* (#F)

Seeing midwives in charge from a variety of racial and ethnic backgrounds was also empowering:

*‘I was ecstatic to see so many midwives of color with such advanced positions.’* (#G)

Some US students felt that UK midwifery was limited by its focus on pregnancy and birth rather than the entire reproductive life span:

*‘I am thankful for my training in primary care and gynecology. The UK limits midwives to a small area of women’s health in comparison.’* (#A)

However, it was evident to students that the healthcare system in the UK promoted midwifery autonomy in a way that the US does not:

*‘The US also creates barriers for midwives in the form of private health insurance. Midwives must struggle against insurance companies to be compensated for routine services. In the UK, midwives are employed by the National Health Service and have limited billing concerns.’* (#A)

Reflections on midwifery autonomy in the UK inspired students to consider political advocacy when they returned to the US:

*‘This trip also lit a fire in me to be an advocate for policy change in the US so that CNMs have the autonomy the registered midwives have in the UK.’* (#D)

These US students embarking on their last term in their education were aware of the impact of regulations on midwifery bedside practice.

### Post-trip themes

*Theme: Goals met and influenced*

In the post-trip narratives, time and distance from the experience allowed reflection. Students expressed that their learning goals for the trip were met. Enhanced confidence was noted as a positive outcome that helped in shaping their ideas and career aspirations:

*‘The STSA experience has influenced my career goals by informing me of midwifery practices abroad that can be utilized in the US. This experience motivates me to become active in support of efforts to increase the midwifery workforce, autonomy, and training of midwives.’* (#C)

*Subtheme: The kind of midwife I want to become*

Students reflected on the influence of this STSA on their developing midwifery identity:

*‘This experience has caused me to think really hard about the kind of midwife I want to become.’* (#F)

Students reported a desire to incorporate some of the systems-level policy changes they felt enabled midwifery to thrive in the UK, such as respect for midwifery, integrated support for community birth, and common care practices, such as easy access to waterbirth, mobility in labor, outpatient cervical ripening, and intermittent auscultation fetal monitoring.

Several language differences resonated with the US students and influenced their future goals. Terms describing labor and birth as ‘straightforward’ or ‘complex’ were viewed favorably compared to the US terms of ‘low-risk’ and ‘high-risk’. The word ‘risk’ implies danger and has the unintended effect of pathologizing birth for providers and clients alike. US students preferred the UK language, which promoted a more balanced perspective on childbirth. UK phrasing that supported autonomy when discussing care options was noticed. UK midwives consistently used the term ‘recommend’ as opposed to the more common US terms of ‘manage’ or ‘order’. Several US students intended to use the UK language choices in their practice.

Post-trip narratives demonstrated an overall positive assessment of the STSA experience that helped shape the kind of midwife students aspired to become:

*‘This short-term study abroad program broadened my global understanding of midwifery care and allowed me to see midwives through the eyes of another culture. The experience provided an example of the role and importance of midwives in supporting communities.’* (#A)

## DISCUSSION

This study reflects learning from an STSA program in which ten US midwifery students traveled to the UK for an immersive experience in a country where midwifery care is the standard of maternity care. Students were seeking another viewpoint from which to understand and gain confidence in their chosen profession, which motivated their participation. Once in the country, they had eye-opening experiences that culminated in meeting their learning goals and developing a sense of new possibilities for their burgeoning midwifery identity. These adult learners are transitioning to include a professional identity as a midwife. The formation of a professional identity is shaped by individual agency and socializing experiences^20^. An STSA in a country where midwifery is a mainstream and valued profession can be helpful to participants’ professional identity formation as a midwife. Upon their return, narratives affirmed that goals for the trip were met and the experience influenced their goals for their career. The study added unique knowledge to the influence of STSAs on student midwives learning.

As students prepared for their UK experience, new perspectives on midwifery care were evolving through preparatory materials, particularly in comparison to the US system. ‘Another viewpoint’ was affirmed during the trip and upon reflection. Students anticipated growth in their professional confidence in the subtheme ‘Strengthen my confidence’. This was a reported outcome as students observed diverse UK midwives in leadership roles in practice, teaching, and administration, sharing their knowledge and experience. This outcome is consistent with other studies exploring the impact of STSAs^14,16^.

The ‘Eye-opening’ subthemes: ‘Respect for midwifery’ and ‘Midwifery autonomy’, reflected students’ understanding of the differences in the status of midwifery in the US and the UK. Midwives were seen as more respected and autonomous in childbirth care in the UK, while US midwives had a highly valued expanded scope of practice. ‘Elevate the bed’ reinforced respect for the knowledge of UK midwives about physiological birth, leading to rooms being structurally arranged to support that knowledge. Negative student reactions to open labor wards, or their dismay that UK midwives were unaware of US midwifery practice, suggested unconscious American (US) ethnocentrism. Still, students displayed cultural humility and appreciation in reflecting a desire to take home practices and attitudes from the UK to use in their future careers.

Critical reflection on the experiences of an STSA has been shown to be an important aspect of the value of the experience^15^. The daily diary entries and post-trip narrative prompts were designed to facilitate such reflection. Students felt their learning goals were achieved in ‘Goals met and influenced’, occurring as new ways of being a midwife in a different system were introduced. The short-term impact of these reflections on their future practice in ‘The kind of midwife I want to become’ were intentions to use language modeled in the UK that implied shared decision-making with clients, rather than medical dominance, in clinical decision-making. Additionally, small changes in the environment, such as elevating the bed, were relatively easy ways to facilitate physiological labor and birth in the US hospital environment. Students were eager to apply these practice changes in their clinical rotations and as practicing midwives. There is potential for students to advocate for and create MLUs as they move into their careers since they have seen that midwifery clinical leadership can work within hospital systems and can achieve positive outcomes.

Students in healthcare professions often engage in international trips to gain experience in hands-on clinical care^21^. Many of these learning opportunities have involved students in high-income countries traveling to low-income countries. Ethical concerns arise when differences in power and privilege exist between hosts and guests. Connecting with similar countries for systems learning can mitigate power and privilege differentials. However, educational experiences in these circumstances must still be developed with an eye for equitable collaboration and respectful communication.

This STSA was structured to look at systems-level issues that affect clinical care in a country where midwifery care is more common and childbirth outcomes are more positive than in the US. Clinical learning can occur in STSAs that provide exploration of systems and facilities and discussions with peer healthcare providers. Learning through sharing and storytelling is a time-honored pedagogical practice in midwifery. These experiences contributed to a powerful immersion experience for students.

### Educational implications

Cultural curiosity and humility should be part of pre-trip preparation for visitors and hosts alike^22^. While the US students had assigned learning activities prior to travel, the UK students did not. Communication with an internet pen-pal was voluntary and arose organically between the student pairs. US students did note a lack of understanding and curiosity regarding US midwifery practice by their UK hosts. Given their place in developing their professional identity, US students were eager to share. A more structured pre-trip preparation for visitors and hosts is suggested. Additionally, integrating international experiences into midwifery curricula can foster professional confidence and shape midwifery identity and should be supported. Making funds available to ease cost concerns and embedding assignments related to the STSA experience into existing courses are ways that academic institutions can promote this type of learning experience.

### Strengths and limitations

The male and gender-diverse midwife student perspective is missing from this study. This STSA was open to a particular cohort of students who self-selected to participate. Students’ ability to fund the trip or manage other personal obligations affected those who chose to travel, potentially influencing study findings. This educational opportunity was structured to call attention to MLUs and a care practice of the student’s choice compared to the US. In doing so, other salient features of the experience may not have received the same level of focus and reflection. This study addresses the gap in the literature by focusing on midwifery students’ experiences of STSAs. These qualitative findings contribute by explicating the meanings of this STSA to midwifery students and may provide insight into the STSA experiences of other types of students.

Research is needed to describe clinical and cultural learning outcomes of STSAs for midwifery students, exploring the development of the midwifery role and identity of participants. The long-term career influence on those midwives who engage in such experiences should be examined. Given the need for midwife consultants worldwide, the question of whether STSAs inspire future work in global health should be explored.

## CONCLUSIONS

Exposing US midwifery students to midwifery care in other countries can support a transformative approach to tackle some of the social, political, and economic issues that constrain midwifery in their home communities. Such experiences can facilitate reflection on how their preconceived ideas, assumptions, and understanding of midwifery practice can be informed and enriched through STSA learning opportunities. Seeing themselves as part of a larger global community of midwives can position US midwifery students to advocate for the system-level changes necessary to address the crisis in maternal outcomes in the US and abroad.

## Data Availability

The data supporting this research are available from the authors on reasonable request.
